# Accurate Prediction of Protein Functional Class From Sequence in the *Mycobacterium Tuberculosis* and *Escherichia Coli* Genomes Using Data Mining

**DOI:** 10.1002/1097-0061(200012)17:4<283::AID-YEA52>3.0.CO;2-F

**Published:** 2000

**Authors:** Ross D. King, Andreas Karwath, Amanda Clare, Luc Dehaspe

**Affiliations:** 1 Department of Computer Science University of Wales Aberystwyth, Penglais Aberystwyth Ceredigion SY23 3DB UK; 2 PharmaDM Celestijnenlaan 200A Leuven B-3001 Belgium

## Abstract

The analysis of genomics data needs to become as automated as its generation. Here we present a novel data-mining approach to predicting protein functional class from sequence. This method is based on a combination of inductive logic programming clustering and rule learning. We demonstrate the effectiveness of this approach on the *M. tuberculosis* and *E. coli* genomes, and identify biologically interpretable rules which predict protein functional class from information only available from the sequence. These rules predict 65% of the ORFs with no assigned function in *M. tuberculosis* and 24% of those in *E. coli*, with an estimated accuracy of 60–80% (depending on the level of functional assignment). The rules are founded on a combination of detection of remote homology, convergent evolution and horizontal gene transfer. We identify rules that predict protein functional class even in the absence of detectable sequence or structural homology. These rules give insight into the evolutionary history of *M. tuberculosis* and *E. coli*.


**Yeast**
*Yeast* 2000; **17**: 283–293.

**Research Article**

### Accurate prediction of protein functional class from sequence in the *Mycobacterium tuberculosis* and *Escherichia coli* genomes using data mining

Ross D. King[1]*, Andreas Karwath[1], Amanda Clare[1] and Luc Dehaspe[2]

[1] *Department of Computer Science, University of Wales, Aberystwyth, Penglais, Aberystwyth, Ceredigion SY23 3DB, UK*
[2] *PharmaDM, Celestijnenlaan 200A, Leuven B-3001, Belgium*

**Correspondence to:*
*R.D. King, Department of Computer Science, University of Wales, Aberystwyth, Penglais, Aberystwyth, Ceredigion SY23 3DB, UK.*

Received: 21 June 2000
Accepted: 12 October 2000

### Abstract

**The analysis of genomics data needs to become as automated as its generation. Here we present a novel data-mining approach to predicting protein functional class from sequence. This method is based on a combination of inductive logic programming clustering and rule learning. We demonstrate the effectiveness of this approach on the *M. tuberculosis* and *E. coli* genomes, and identify biologically interpretable rules which predict protein functional class from information only available from the sequence. These rules predict 65% of the ORFs with no assigned function in *M. tuberculosis* and 24% of those in *E. coli*, with an estimated accuracy of 60–80% (depending on the level of functional assignment). The rules are founded on a combination of detection of remote homology, convergent evolution and horizontal gene transfer. We identify rules that predict protein functional class even in the absence of detectable sequence or structural homology. These rules give insight into the evolutionary history of *M. tuberculosis* and *E. coli*. Copyright © 2000 John Wiley & Sons, Ltd.**

**Keywords:** **machine learning; clustering; ILP; bioinformatics**

### Introduction

The genomes of around 30 microorganisms have now been completely sequenced (**Magpie: http://www-fp.mcs.anl.gov/~gaasterland/genome.html**; Blattner *et al.*, 1997; Cole *et al.*, 1998; Goffeau *et al.*, 1996) as have those of the multicellular animals *Caenorhabditis elegans* (*C. elegans* Sequencing Consortium, 1998) and *Drosophila melanogaster* (Adams *et al.*, 2000). This new knowledge is revolutionizing biology. Perhaps the most important revelation from the sequenced genomes is that the functions of only 40–60% of the predicted genes are known with any confidence. For example, in *Saccharomyces cerevisiae*, one of the most intensely studied organisms, of the ca. 6000 predicted protein-encoding genes (Goffeau *et al.*, 1996), the function of only ca. 60% can be assigned with any confidence. The new science of *functional genomics* (Hieter and Boguski, 1997; Bussey, 1997; Bork *et al.*, 1998; Brent, 1999; Dyer *et al.*, 1999) is dedicated to determining the function of the genes of unassigned function and to further detailing the function of genes with purported function.

To meet the challenge posed by functional genomics, new and highly ingenious experimental techniques have been developed to analyse gene function. These techniques permit large-scale and parallel interrogation of cell states under different stages of development and under particular environmental conditions. Such analyses may be carried out at the level of transcription using hybridization arrays (Lockhart *et al.*, 1996; DeRisi *et al.*, 1997; Brown and Botstein, 1999; Alizadeh *et al.*, 2000). Similar analyses may be carried out at the level of translation to define the proteome (Wilkins *et al.*, 1997; O'Connor *et al.*, 1998; Blackstock and Weir, 1999). Most recently, the metabolome (Oliver, 1997;

Copyright © 2000 John Wiley & Sons, Ltd.

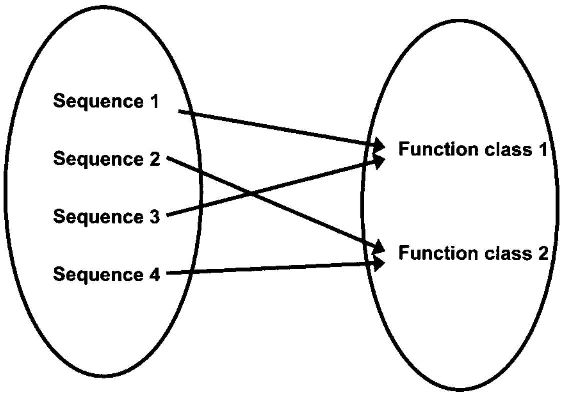

**Figure 1.** Mapping of sequences into functional groups

Oliver and Baganz, 1998; Johnson *et al.*, 2000) and large-scale phenotyping (Rieger *et al.*, 1999) have emerged as other important approaches to functional genomics.

Bioinformatics can greatly facilitate such efforts by making accurate *in silico* predictions of gene function based on nucleotide or residue sequence alone. Such predictions make experimental determination of function simpler, as it is clearly more efficient to test a high probability hypothesis than to randomly test for possible functions. To predict protein function directly from sequence, what is abstractly required is a computable discrimination function (Mitchell, 1997), which maps sequence to biological function (Figure 1). The existing sequence homology recognition methods can be viewed as examples of such functions: methods based on direct sequence similarity (Pearson and Lipman, 1988; Altschul *et al.*, 1997) can be considered as nearest neighbour-type functions (Duda and Hart, 1973) (in sequence space), and the more sophisticated homology recognition methods based on motifs/profiles (Taylor, 1998) resemble case-based learning functions (Aha *et al.*, 1991). The question naturally arises whether there exist other, perhaps more general, types of discrimination function? Given the complexities of the relationship between protein sequence and structure, this would *a priori* seem unlikely. However, the natural way to identify such functions would be to learn them empirically from the annotated sequence databases using data mining techniques (Piatetsky-Shapiro and Frawley, 1991; Chatfield, 1995; Fayyad *et al.*, 1996; Munakata,1999).

To test the hypothesis that data mining could be used to find general types of discrimination functions for predicting function, we focused in on the use of protein functional hierarchies. We believe the recognition of the value of such hierarchies to be one of the most important conceptual advances in functional genomics (Riley & Labedan, 1996). An example of such a hierarchy is that for *M. tuberculosis* taken from the Sanger Centre: **TB_gene_list: http://www.sanger.ac.uk/Projects/M_tuberculosis/gene_list_full.shtm** (Figure 2). In this hierarchy, the protein L-fuculose phosphate aldolase (Rv0727c, fucA) has a top-level class assignment 'small-molecule metabolism', a second-level class

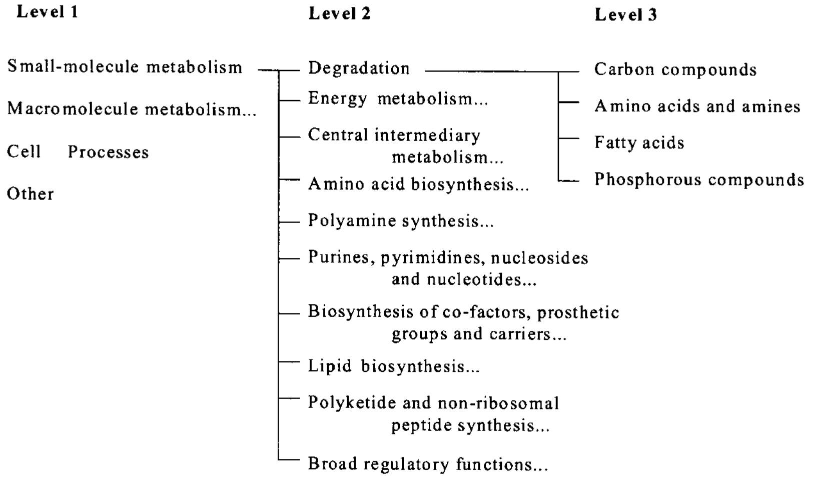

**Figure 2.** An example subset of the genes functional hierarchy in *M. tuberculosis*. The gene L-fuculose phosphate aldolase is in the Level 3 class 'carbon compounds'. This example has only three out of four possible classification levels

Copyright © 2000 John Wiley & Sons, Ltd.

'degradation', and a third-level class 'carbon compounds' (see Figure 2). Such hierarchies are generally either in a strict tree e.g. (the Sanger Centre's *M. tuberculosis* **TB_gene_list**; and Monica Riley's *E. coli* **EC_gene_list**: **http://genprotec.mbl.edu:80/start**), or as directed acyclic graphs DAGs (Kell and King, 2000), e.g. MIPS *Saccharomyces cerevisiae* **SC_gene_list: http://www.mips.biochem.mpg.de/proj/yeast/catalogues/index.html** (It is significant that these hierarchies bear a close resemblance to engineering diagrams showing the hierarchical organization of systems, e.g. in a motorbike the electrical subsystem, the braking subsystem, etc. This way of organizing knowledge goes back to Aristotle.) These hierarchies are important because they provide frameworks for structuring our knowledge of the components of cells and so provide the grouping together of functionally related proteins. The existence of such groupings (classes) opens up the possibility of generalizing over the objects in the class (induction: finding something generally true about the objects in a class). *The novel approach taken in this paper is to find, using data mining, general properties that are true of the sequences in particular functional classes and not true for any other functional class. These properties of the sequence can then be used to predict functional class from sequence.*

### Materials and methods

We selected the genomes of *Escherichia coli* and *Mycobacterium tuberculosis* for study. *E. coli* is probably the best characterized extant genome and is a Gram-negative organism. We used 4289 open reading frames (ORFs) (Blattner *et al.*, 1997) and took the functional assignments from GenProtEC (**EC_gene_list**). *M. tuberculosis*, is a Gram-positive actinomycete and is probably the prokaryote genome of greatest medical importance. We used 3924 ORFs (Cole *et al.*, 1998) and took the functional assignments from the Sanger Centre (**TB_gene_list**). The assignment of function for both organisms is organized in a strict hierarchy (tree), where each higher level in the tree is more general than the level below it, and the leaf nodes are the individual functions of proteins. These functional classes are what we wish to predict from sequence. *The organization of function in M. tuberculosis and E. coli is quite different, reflecting their quite different biology and long evolutionary separation.* We attempted to learn discriminatory functions for every level of the functional hierarchy of both organisms.

The basic methodology for each genome was:

1. *Generate the database*: retrieve the identified ORFs (putative proteins) and the known functional assignments [note that some ORFs will be shown not to code for proteins and there are errors in annotation of function (Brenner, 1999), and this adds 'noise' to the data-mining process (Mitchell, 1997)]; then compute for each ORF in the genome a set of descriptors based solely on information available from the sequence.
2. *Data mining*: learn (induce) rules that map sequence descriptions to function; test these rules on ORFs not used in learning the function.

To generate the database to mine we retrieved for each organism the ORFs and their known functional assignments. *The description of each ORF is only based on features that can be computed from sequence.* The most commonly used technique to gain information about a sequence is to run a sequence similarity search, and this was used as the starting point in forming the descriptions. The basic data structure is based on the result of a PSI-BLAST search. We used the parameters (Altschul *et al.*, 1997): $e=10$, $h=0.0005$, $j=20$, NRProt 16/11/98 for *M. tuberculosis*, and NRProt 05/10/99 for *E. coli*). For each ORF, and for each protein identified as having sequence similarity to it, we formed an expressive description based on: the frequency of singlets and pairs of residues in the protein; the phylogeny of the organism from which the protein was obtained-from SWISS-PROT (Bairoch and Apweiler, 2000); SWISS-PROT keywords (membrane; transmembrane; inner_membrane; outer_membrane; repeat; plasmid; alternative_splicing); the length and molecular weight of the protein, its pI and molecular composition (**ProtParam_tool**: **http://www.expasy.ch/tools/protparam.html**) (only for *E. coli*); and the protein's predicted secondary structure using Prof (Ouali and King, 2000) (only for *E. coli*). This description resembles a 'phylogenic profile' (Marcotte *et al.*, 1999) but is more general. The main differences in describing phylogeny are that the Datalog description includes information on:

Copyright © 2000 John Wiley & Sons, Ltd. 

1. All the species with detected homologies.
2. The actual phylogenic classification of each species – allowing generalization over any level of taxa.
3. The significance (evolutionary distance) of the predicted homology.
4. The sizes of the predicted homologous proteins.
5. Keywords describing the predicted homologous proteins, especially those relating to membrane binding.

For *E. coli*, 10 097 865 facts were generated and 5 895 649 for *M. tuberculosis* (see Table 1 for details).

We mined this database to generate rules that predict protein functional class from sequence description. We used two complementary forms of data mining: inductive logic programming (ILP) (Muggleton, 1991; Lavrac and Dzeroski 1994) and propositional rule learning (Mitchell, 1997). These forms of data analysis differ from traditional statistical methods in that they are based on using a symbolic language to describe the examples and inductive theories formed. In propositional rule learning the language used is that of attribute vectors (propositional logic). A characteristic of attribute vectors is that all the information about a particular example can be put into a single row of a table. Rules (theories) are of the form: **if** a conjunctions of conditions (attributes having certain values) is true for an example, **then** the example belongs to a particular class. For example, a rule for classifying bacteria is: **if** 'resistant to gamma radiation' = true **and** 'cell wall contains ornithine' = true, **then** genus = *Deinococcus*. Such rules are called 'propositional classification rules'. An efficient way of learning such rules is to first learn an intermediate structure known as a decision tree. In a decision tree, each node is a test of an attribute and the leaves are classes. It is in general computationaly infeasible to learn an optimal decision tree, but greedy approaches based on recursively choosing the best attribute test have been shown to be very effective (Mitchell, 1997). We used the popular C4.5 and C5 decision tree-based methods for learning propositional rules (Quinlan, 1993).

ILP uses the rich language of logic programs to describe examples and theories. This language is more expressive than that of attribute vectors (propositional logic). Logic programs are based on first-order predicate logic and are equivalent in expressive power to standard computer languages, such as Fortran and Java. This greater expressive power allows ILP to find solutions to problems that cannot be solved using standard statistical or neural network techniques (which are based on attribute vectors), and enables results to be learnt that are more human-understandable (King *et al.*, 1992; Lavrac and Dzeroski 1994; King *et al.*, 1996).

We used the ILP data-mining programme WARMR (Dehaspe *et al.*, 1998). This programme is designed for the prototypical data mining task of finding *all* frequently occurring patterns of a particular type. WARMR employs an efficient levelwise method similar to the Aprior algorithm (Fayyad *et al.*, 1996), which allows it to be used on very large databases. The algorithm is based on a breadth-first search of the pattern space (which is ordered by the generality of patterns) (Mannila and Toivonen, 1997). Pruning is based on the monotonicity of specificity with respect to frequency – if a pattern is not frequent then none of its specializations can be frequent. This learning method allows fast and efficient on large databases.

The combined ILP propositional data-mining methodology used was as follows (see Figure 3):

1. *Clustering*: randomly select 2/3 of the ORFs as training data and 1/3 as test; run WARMR on the training data to identify frequent patterns in the descriptions (e.g. a frequent pattern was the occurrence of the keyword 'transmembrane' in high molecular weight proteins); convert the identified frequent patterns to Boolean indicator attributes (i.e. if an ORF has the above frequent pattern, then a particular attribute has the value *true*: if this patterns is not present, then the attribute has the value *false*).
2. *Rule learning*: randomly select 1/3 of the ORFs in the training data as validation data; use C4.5 or C5 on the training data (excluding the validation data), to learn rules that predict function from the descriptional attributes; select the best learnt prediction rules on the basis of their performance on the validation data; and test their performance on the test data.
3. *Prediction*: apply the prediction rules to ORFs which have not been assigned a function.

Rules were selected to balance accuracy with unidentified gene coverage. *For any application, the correct balance of accuracy and coverage depends on the relative cost of making errors of commission and*

Copyright © 2000 John Wiley & Sons, Ltd.

Table 1. The basic descriptors used to describe ORFs

| Descriptor | Explanation |
|---|---|
| amino_acids_R | The number of residues of type **R** in the sequence |
| amino_acid_ratio_R | The percentage composition of residues of type **R** |
| amino_acid_pairs_RS | The number of residue pairs of type **R**, **S** in the sequence |
| amino_acid_pair_ratio_RS | The percentage composition of residue pairs of type **R**, **S** |
| sequence_length | The number of residues in the sequence |
| molecular_weight | The computed molecular weight |
| aliphatic_index | The computed aliphatic index |
| hydro | The grand average of hydropathicity (GRAVY); the value was discretized, 1 for low values, increasing up to 5 for high values |
| PI | The theoretical isoelectric point (pI) for this ORF |
| atomic_comp_E | The ORF's atomic composition of element **E**; where **E** is one of the following: carbon (C), hydrogen(H), nitrogen(N), oxygen(O) or sulphur(S) |
| hom(P) | **P** is a homologous protein found by PSI-BLAST |
| e_val_rule(P, E) | **P** is a homologous protein found by PSI-BLAST with sequence similarity measure **E** |
| e_val_lteq(P, X) | **P** is a homologous protein found by PSI-BLAST with sequence similarity measure less than **X** |
| e_val_gt(P, X) | **P** is a homologous protein found by PSI-BLAST with sequence similarity measure greater than **X** |
| psi_val_rule(P, It) | **P** is a homologous protein found by PSI-BLAST on iteration **It** |
| psi_iter_lteq(P, X) | **P** is a homologous protein found by PSI-BLAST on iteration less than **X** |
| psi_iter_gt(P, X) | **P** is a homologous protein found by PSI-BLAST on iteration greater than **X** |
| species(P, Species) | The protein **P** comes from species **Species** |
| classification(P, Class) | The protein **P** comes from a species with SwissProt phylogenic classification **Class** |
| mol_wt_rule(P, X) | The protein **P** has discretized molecular weight **X** |
| mol_wt_lteq(P, X) | The molecular weight of **P** is less than **X** |
| mol_wt_gt(P, X) | The molecular weight of **P** is greater than **X** |
| keyword(P, Word) | The SwissProt keyword **Word** describes protein **P** |
| ss(S, T) | Position **S** is predicted to be a secondary structure element of type **T** |
| nss(S1, S2, T) | Given the secondary structure at position **S1**, the neighbouring position **S2**, with **S2 = S1 + 2**, has a secondary structure prediction of type **T** |
| ss_alpha(S, gt, X) | Position **S** is predicted to be an alpha-helix of length greater than **X** (similarly lteq instead of gt) |
| ss_beta(S, gt, X) | Position **S** is predicted to be a beta-strand of length greater than **X** (similarly lteq instead of gt) |
| ss_coil(S, gt, X) | Position **S** is predicted to be a coil of length greater than **X** (similarly lteq instead of gt) |
| nss_alpha(S1, S2, gt, B) | Positions **S1** and **S2** (where **S2 = S1 + 2**) are predicted to be alpha-helices of length greater than **X** (similarly lteq instead of gt) |
| nss_beta(S1, S2, gt, X) | Positions **S1** and **S2** (where **S2 = S1 + 2**) are predicted to be alpha-helices of length greater than **X** (similarly lteq instead of gt) |
| nss_coil(S1, S2, gt, X) | Positions **S1** and **S2** (where **S2 = S1 + 2**) are predicted to be alpha-helices of length greater than **X** (similarly lteq instead of gt) |

The descriptors above the bold line are propositional. X is an amino-acid residue; there are considered to be 21 residues, the standard 20 plus x (for repetitive sequences). The descriptors: aliphatic_index, hydro, pI, and atomic_comp_E were generated using the **ProtParam** programme. The values described in the table by 'X' are discretized into five classes (1 very low, 2 low, 3 medium, 4 high, and 5 very high). The E value of a PSI-BLAST search is a measure of the probability of a sequence match being homologous (note that a low value means a high sequence similarity); it can also be considered as a measure of evolutionary relatedness of the homologous protein. For secondary structure descriptors, positions refer to the order in the predicted secondary structure. If, for example, an ORF has the following predicted secondary structure: $\alpha\alpha\alpha\alpha cccccc\alpha\alpha\alpha\alpha\alpha ccccccc\beta\beta\beta$ would translate into: the 1st alpha-helix secondary structure prediction is of length 4; the 1st coil secondary structure prediction is of length 6; the 2nd alpha-helix secondary structure prediction is of length 5; the 2nd coil secondary structure prediction is of length 7; and the 1st beta-strand structure prediction is of length 3.

*omission* (Provost and Fawcett, 1997) (making incorrect predictions vs. missing genes). The system can be tuned to select different balances. The prediction rules were then applied to genes that have not been assigned a function.

### Results and discussion

For both *M. tuberculosis* and *E. coli* it was possible to find good rules that predict function from sequence at all levels of the functional hierarchies

Copyright © 2000 John Wiley & Sons, Ltd.

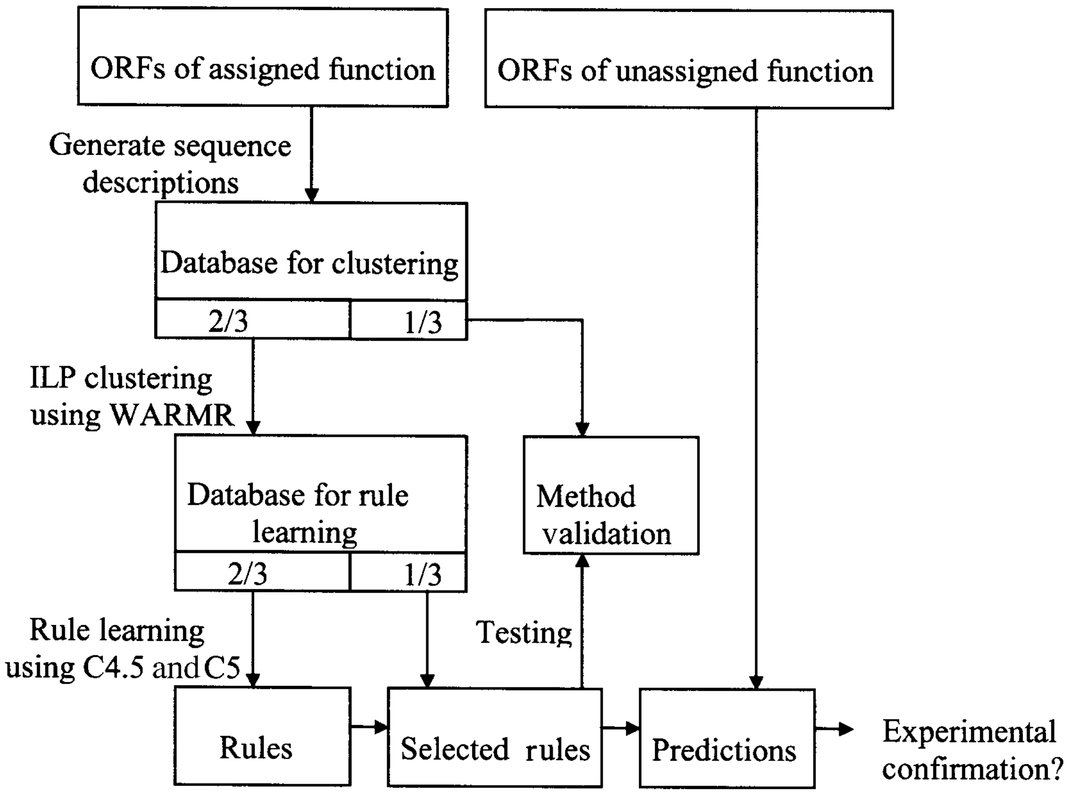

**Figure 3.** Flow chart of the data mining methodology. This hybrid approach has proved successful in the past on other scientific discovery tasks (Dehaspe *et al.*, 1998). It is powerful because the clustering improves the representation for learning (using the expressive power of ILP) and the discrimination step efficiently exploits the pre-labelled examples. Good rules were selected on a validation set and the unbiased accuracy of these rules estimated on a test set

Table 2. Learning results for *M. tuberculosis* and *E. coli*

| | *M.tuberculosis* | | | | *E. coli* | | |
|---|---|---|---|---|---|---|---|
| | *Level 1* | *Level 2* | *Level 3* | *Level 4* | *Level 1* | *Level 2* | *Level 3* |
| Number of rules found | 25 | 30 | 20 | 3 | 13 | 13 | 13 |
| Rules predicting more than one homology class | 19 | 18 | 8 | 1 | 9 | 10 | 3 |
| Rules predicting a new homology class | 14 | 15 | 1 | 0 | 9 | 5 | 3 |
| Average test accuracy | 62% | 65% | 62% | 76% | 75% | 69% | 61% |
| Default test accuracy | 48% | 14% | 6% | 2% | 40% | 21% | 6% |
| New functions assigned | 886 (58%) | 507 (33%) | 60 (4%) | 19 (1%) | 353 (16%) | 267 (12%) | 135 (6%) |

The number of rules found are those selected on the validation set. A rule predicts more than one homology class if there is more than one sequence similarity cluster in the correct test predictions. A rule predicts a new homology class if there is a sequence similarity cluster in the test predictions that has no members in the training data. Average test accuracy is the accuracy of the predictions on the test proteins of assigned function (if conflicts occurred, the prediction with the highest *a priori* probability was chosen). Default test accuracy is the accuracy that could be achieved by always selecting the most populous class. 'New functions assigned' is the number of ORFs of unassigned function predicted. It would have been better to use cross-validation or some similar resampling method (Mitchell, 1997) to estimate the variance in these values; however, this would have been computationally infeasible because of the large size of the databases. The test accuracy estimates may be too pessimistic, as proteins may have more than one functional class but only one of these is considered correct (see examples in the text). However, it is also possible to argue that estimates are too optimistic, as the ORFs of unassigned function come from a different distribution from that used to train the rules. Only by empirically testing the prediction rules can the true accuracy of the rules be determined

Copyright © 2000 John Wiley & Sons, Ltd. 

| | |
|---|---|
| **If** | the percentage composition of lysine in the ORF is > 6.6%, |
| **then** | its functional class is 'macromolecule metabolism' |

**Figure 4.** Rule TB_C50_1_26 a top-level rule from *M. tuberculosis*. This rule is 85% (11/13) accurate on the test set (the probability of this result occurring by chance is estimated at $1.2 \times 10^{-5}$ as the class 'macromolecule metabolism' covers ca. 25% of examples). The rule correctly predicts the following proteins (rpsG (S7), rpsI (S9), rpsL (S12), rpsT (S20), rplJ (L10), rplP (L16), rplS (L19), rplX (L24), rpmE (L31), rpmJ (L36), infC (IF-3)). These proteins are all involved in protein translation. When the training data are included, the rule covers 46 out of the 58 proteins known to be involved in ribosomal protein synthesis and modification. The two errors (of commission) made in the test data were groEL2 and Rv3583c, a 'putative transcriptional regulator'. The rule predicts the function of five ORFs classed as 'conserved hypotheticals' (Rv566, Rv854, Rv910, Rv2185, Rv2708) and 10 ORFs classed as 'unknowns' (Rv123, Rv810, Rv909, Rv1893, Rv1955, Rv2061, Rv2517, Rv2819, Rv2822, Rv3718). The prediction rule is consistent with protein chemistry, as lysine is positively charged which is desirable for interaction with negatively charged RNA. The choice of lysine over arginine for the positively charged residue may be connected with the high GC content of the *M. tuberculosis* genome (2) – lysine is coded by the codons AAA and AAG while arg is coded by CGU, CGC, CGA and CGG

(Table 2). The test accuracy of these rules is far higher than possible by chance. In *M. tuberculosis*, of the ORFs originally in the 'conserved hypothetical' or 'unknown' function classes, 985 (65%) were predicted to have a function at one or more levels of the hierarchy. In *E. coli*, of the ORFs with no assigned function ('unknown function' and 'miscellaneous' classes), 525 (24%) were predicted to have a function at one or more levels of the hierarchy. The rules are estimated to have accuracies in the range 60–80%. The rule learning data, the rules and the predictions are all given at the site: **http://www.aber.ac.uk/~dcswww/Research/bio/ProteinFunction/**. We illustrate the value of the rules by describing, in Figures 4, 5 and 6, three of the rules learnt.

The number of these predictions varies by level in the functional hierarchy, with most predictions being made at the top level. We found this surprising as, at the start of the investigation, we did not believe it would be possible to find rules that recognized such broad functional classes as, for example, between 'small molecule metabolism' and 'macromolecule metabolism'. The most valuable predictions are those at the lower levels, as these can be tested most easily experimentally. At this

**Figure 5.** Rule TB_C50_3_3 is a level 3 rule for *M. tuberculosis*. This rule is 80% (16/20) accurate on the test set (the probability of this result occurring by chance is $4.8 \times 10^{-18}$). It correctly predicts nine acyl-CoA synthases (fadD6, fadD9, fadD10, fadD11, fadD15, fadD18, fadD25, fadD26, fadD34), six enoyl-CoA hydratases (echA4, echA14, echA15, echA16, echA18, echA19), and the fatty-acid $\beta$-oxidation complex $\alpha$-subunit (fadB) which is homologous to enoyl-CoA hydratases. The acyl-CoA synthases and enoyl-CoA hydratases are not thought to be homologous. The four errors (of commission) made by this rules were: pks16 (a possible polyketide synthase); sucD (succinyl-CoA synthase alpha chain); nrp (an unknown non-ribosomal peptide synthase); and mbrF (mycobactin/exochelin synthesis). It is interesting that two of these errors, sucD and pks16, are involved in lipid metabolism. Although the prediction rule is quite complicated and non-intuitive, the rule clearly is effective and cannot be explained by chance; it must therefore represent some real biological regularity

Copyright © 2000 John Wiley & Sons, Ltd. 

**If** the ORF is not predicted to have a β-strand of length ≤ 3 ∧

a homologous protein from class Chytridiomycetes was found

**then** function is 'cell processes, transport/binding proteins'

**Figure 6.** Rule EC_C50_2_CSH_44, a second level *E. coli* rule. This rule is 86% (12/14) accurate on the test set (the probability of this result occurring by chance is estimated at $4 \times 10^{-7}$ as the class 'transport/binding proteins' covers ca. 16% of examples). The rule correctly predicts the following proteins: gntT, b1514, ugpE, ytfT, livH, yebI, hisC, b2546, yjgT, yejB, codB, b0831. These are transport proteins currently mainly classified into the ABC superfamily. Its errors (of commission) are: nrfD, which is classed as 'energy metabolism, carbon' but has 'a putative STP transport domain' (illustrating the problem of assigning only one functional class per protein); and cyoE, which may also have a transport role. The rule predicts 24 ORFs of unassigned function: b0007, b0155, b0328, b0371, b0787, b0788, b0790, b0813, b0818, b1515, b1688, b1752, b2317, b2365, b2578, b2689, b3009, b3071, b3151, b3522, b3819, b4210, b1599, b4262. The rule is based on a mixture of structural and phylogenic data. Analysis shows that homology to the *Chytridiomycetes* mitochondrial protein cytochrome *c* oxidase (polypeptide I) is important. The structural attribute selects the transport proteins from other homologues. The significance of the primitive fungi class *Chytridiomycetes* is unclear level of detailed prediction it is relatively easy to envisage experiments to confirm the predictions. For example, in *M. tuberculosis* ORF Rv2752 is predicted to have the functional class 'small-molecule metabolism, degradation, fatty acids'. If this ORF was knocked out and the fatty acid composition of the organism changed, then this would be consistent with the hypothesis. The rule learning data, the rules and the predictions, are given at **http://www.aber.ac.uk/~dcswww/Research/bio/ProteinFunction/**.

Perhaps the most convincing evidence for the ability to predict function from sequence is revealed by analysis of some of the errors made. For example, rule TB_C50_3_6 is a level three rule for *M. tuberculosis* which predicts the functional class 'small-molecule metabolism, energy metabolism, miscellaneous oxidoreductases and oxygenases'. It is 60% accurate (6/10) on the test set, and the four errors of commission are: nirB [nitrite reductase flavoprotein], fabG1 [3-oxoacyl-(ACP) reductase], fabG3 [3-oxoacyl-(ACP) reductase], and amiD

Table 3. Comparison of the use of ILP (Warmr) data mining on *E. coli*

| Method | Level 1 | Level 2 | Level 3 |
|---|---|---|---|
| Accuracy: propositional | 64% | 63% | 41% |
| Accuracy: propositional + ILP | 75% | 69% | 61% |
| Unassigned ORFs: propositional | 359 | 245 | 63 |
| Unassigned ORFs: propositional + ILP | 353 | 267 | 135 |

The accuracies are the test set accuracies and the unassigned ORFs predicted are those not annotated in **EC_gene_list**.

[probable amidase]. Of these nirB, fabG1 and fabG3 are clearly reductases, and amiD is a related enzyme. It is very hard to see how such biologically meaningful 'errors' could have arisen by a systematic methodological error. This highlights the generic problem that existing functional hierarchies often give only one function per gene (Kell and King, 2000).

To test the value of using the ILP step in the learning process, we compared results with and without ILP on *E. coli* (Table 3). The table shows that using the ILP descriptors increases both the estimated accuracy of the rules learnt and their coverage of unannotated ORFs.

For those proteins correctly predicted by each rule, we carried out all-against-all PSI-BLAST searches. If all the proteins could be linked together by PSI-BLAST scores < 10, then the proteins were considered homologous (this is a very liberal definition). We found that many of the predictive rules were more general than possible using sequence homology. This was shown in two ways: the rules correctly predict the function of sets of proteins that are not homologous to each other; and they correctly predict the function of proteins that are not homologous to any in the training data (Table 2). *Such rules provide a way of predicting function in the absence of recognizable sequence homology.*

The other rules were based on homology. Although these rules are not doing anything that could not be done in principle by existing homology prediction methods, they are still valuable. They provide a novel way of detecting homology, and this is one of the most important processes in bioinformatics. It is to be expected that, combined with standard homology detection programmes such as PSI-BLAST, the prediction rules could

Copyright © 2000 John Wiley & Sons, Ltd. 

predict more distant homologies than existing methods alone.

The rules discovered are important in two ways: they make predictions that are useful in determining the functions of ORFs of currently unknown function, and they provide evolutionary insight. The actual function of an ORF can only be determined by 'wet' experiment. However, bioinformatic techniques, such as sequence homology detection, and the prediction rules presented here, can make such experimental determination simpler. We look forward to the testing of our predictions by other workers, and we are designing automatic methods to test the rules ourselves.

The existence of general rules for predicting biological function raises the question of their evolutionary causation. How are such rules possible, given the notoriously complicated mappings between function and structure, and structure and sequence? Several possibilities exist: the rules are paralogous (Henikoff *et al.*, 1997; Tatusov *et al.*, 1997), with homology so distant as to be undetectable by sequence analysis; or convergent evolution has occurred, forcing proteins with similar function to resemble each other; or horizontal evolution has transferred functional related groups of protein into the organisms. Evidence in favour of a role for distant homology is that it was possible to predict function better than random, based on predicted secondary structure alone; and secondary structure is better conserved over evolution than sequence (Park *et al.*, 1997). Evidence against this is that we have found little evidence for common SCOP database (Murzin *et al.*, 1995) 'superfamily' and 'fold' classifications for proteins predicted by the same rule. Convergent evolution seems to be the dominant factor in rules such as *M. tuberculosis* rule TB_C50_1_26 (Figure 4). Evidence for horizontal transfer of genes into *M. tuberculosis* and *E. coli* is the importance of phylogeny in many rules where a paralogous explanation seems to be ruled out.

One limitation with the current approach is that many of the rules, despite being accurate, are often quite complicated (e.g. Figure 5), and their biological basis comparatively difficult to understand. It is to be hoped that refinements in the background knowledge used to describe the sequences will remove some of these problems, although it would be naive to expect extremely simple explanatory rules for all functional classes. Improvements in the background knowledge used for learning and the learning techniques should also increase the accuracy and coverage of the predictions. At present only some of the functional classes can be predicted, which may be partly a reflection on that these classes are more natural or appropriate. There is also a pressing need for the semantics of what is meant by a 'function' to be made explicit in functional genomics. Some relevant work has been done in this area in engineering (Chittaro *et al.*, 1993). One important feature to note about the current rules is that they are time-dependent. For example, in Figure 6, if more sequences become known from *Chytridiomycetes*, then the rule would not necessarily be valid. In such cases it is important to distinguish between the inference rule and the likely causation of the regularity in the data; prediction rules need not be causative (Jaynes, 1994).

The rules presented in this paper are organism-specific (either for *M. tuberculosis* or *E. coli*); however, this is not an essential feature of the method. We learnt such rules because there does not exist a single consistant functional hierarchy that encompasses both *M. tuberculosis* and *E. coli*. This is partly due to the radically different biology in the two species, requiring different functional classes (King and Kell, 2000). It is also due to a lack of coordination in sequence annotation. This latter problem has now been recognized and work has started on controlled vocabularies and ontologies (e.g. **Ontology**: **http://www.geneontology.org/**). When such ontological work has annotated a sufficient number of species, it will be possible to search for pan-specific rules relating sequence to functional class.

The approach taken in this paper to predicting biological function is complementary to those using data mining to analyse other forms of bioinformatic data, such as expression profiles, pathway analysis, structural studies, etc. Information from these diverse sources will be able to be combined together to produce more powerful predictions than any single one in isolation.

### Acknowledgements

We would like to thank Douglas B. Kell and Michael Young from IBS, and Mohammed Ouali from Computer Science, at the University of Wales, Aberystwyth; Ashwin Srinivasan of the Computing Laboratory at the University of Oxford; and

Copyright © 2000 John Wiley & Sons, Ltd. 

Steffen Schulze-Kremer at the Resource Centre of the German Human Genome Project, for helpful discussions. This work was supported by the EPSRC, Grant No. BR/L262849; the MRC, Grant No. G78/6609; and the British Council, Grant No. KN/991/11/PRO986/KB-ss.

### References

Adams MD *et al.* 2000. The genome sequence of *Drosophilia melanogaster*. *Science* **287**: 2185–2195.

Aha D, Kibler D, Albert M. 1991. Instance-based learning algorithms. *Machine Learning* **6**: 37–66.

Alizadeh A *et al.* 2000. Distinct types of diffuse large B-cell lymphoma identified by gene expression profiling. *Nature* **403**: 503–511.

Altschul SF *et al.* 1997. Gapped BLAST and PSI-BLAST: a new generation of protein database search programs. *Nucleic Acids Res* **25**: 3389–3402.

Bairoch A, Apweiler R. 2000. The SWISS-PROT protein sequence database and its supplement TrEMBL. *Nucleic Acids Res* **28**: 45–48.

Blackstock WP, Weir MP. 1999. Proteomics: quantitative and physical mapping of cellular proteins. *Tibtech* **17**: 121–127.

Blattner FR *et al.* 1997. The complete genome sequence of *Escherichia coli* K-12. *Science* **277**: 1453–1461.

Bork P, Dandekar T, Diaz-Lazcoz Y *et al.* 1998. Predicting function: from genes to genomes and back. *J Mol Biol* **283**: 707–725.

Brenner E. 1999. Errors in gene annotation. *Trends Genet* **15**: 132–133.

Brent R. 1999. Functional genomics: learning to think about gene expression data. *Curr Biol* **9**: R338–R341.

Brown PO, Botstein D. 1999. Exploring the new world of the genome with DNA microarrays. *Nature Genet* **21**: 33–37.

Bussey H. 1997. 1997 ushers in an era of yeast functional genomics. *Yeast* **13**: 1501–1503.

Chatfield C. 1995. Model uncertainty: data mining and statistical inference. *J R Stat Soc Ser A Stat Soc* **158**: 419–466.

Chittaro L, Guida G, Tasso C, Toppano E. 1993. Functional and teleological knowledge in the multimodelling approach for reasoning about physical systems: a case study in diagnosis. *IEEE Trans Syst Man Cyber* **23**: 1718–1751.

Cole ST *et al.* 1998. Deciphering the biology of *Mycobacterium tuberculosis* from the complete genome sequence. *Nature* **393**: 537–544.

C. elegans Sequencing Consortium. 1998. Genome sequence of the nematode *C. elegans*: a platform for investigating biology. *Science* **282**: 2012–2018.

**Data: http://www.aber.ac.uk/~dcswww/Research/bio/ProteinFunction/.**

Dehaspe L, Toivonen H, King RD. 1998. Finding frequent substructures in chemical compounds. In *Proceedings of the Fourth International Conference on Knowledge Discovery and Data Mining*, Agrawl R, Stolorez P, Piatetsky-Shapiro G (eds). AAAI Press: Menlo Park, CA; 30–36.

DeRisi JL, Iyer VR, Brown PO. 1997. Exploring the metabolic and genetic control of gene expression on a genomic scale. *Science* **278**: 680–686.

Duda R, Hart P. 1973. *Pattern Classification and Scene Analysis*. Wiley: New York.

Dyer MR, Cohen D, Herrling P. 1999. Functional genomics: from genes to new therapies. *Drug Discovery Today* **4**: 109–114.

**EC_gene_list: http://genprotec.mbl.edu:80/start**

Fayyad U, Piatetsky-Shapiro G, Smyth P, Uthurusamy R. 1996. *Advances in Knowledge Discovery and Data Mining*. AAAI/MIT Press; Boston, MA.

Goffeau A *et al.* 1996. Life with 6000 genes. *Science* **274**: 546–567.

Henikoff S *et al.* 1997. Gene families: the taxonomy of protein paralogs and chimeras. *Science* **278**: 609–614.

Hieter P, Boguski N. 1997. Functional genomics: it's all how you read it. *Science* **278**: 601–602.

Jaynes ET. 1994. *Probability Theory: The Logic of Science*. **http://omega.albany.edu:8008/JaynesBook.html.**

Johnson HE, Gilbert RJ, Winson MK *et al.* 2000. Explanatory analysis of the metabolome using genetic programming of simple, interpretable rules. *Genet Progr Evolvable Machines* **1** (in press).

Kell DB, King RD. 2000. On the optimization of classes for the assignment of unidentified reading frames in functional genomics programmes: the need for machine learning. *Trends Biotechnol* **18**: 93–98.

King RD, Muggleton S, Lewis RA, Sternberg MJE. 1992. Drug design by machine learning – the use of inductive logic programming to model the structure–activity relationships of trimethoprim analogs binding to dihydrofolate-reductase. *Proc Natl Acad Sci U S A* **89**: 11322–11326.

King RD, Muggleton SH, Srinivasan A, Sternberg MJE. 1996. Structure–activity relationships derived by machine learning: the use of atoms and their bond connectivities to predict mutagenicity by inductive logic programming. *Proc Natl Acad Sci USA* **93**: 438–442.

Lavrac N, Dzeroski S. 1994. *Inductive Logic Programming: Techniques and Applications*. Ellis Horwood: Chichester.

Lockhart DJ, Dong HL, Byrne MC *et al.* 1996. Expression monitoring by hybridization to high-density oligonucleotide arrays. *Nature Biotechnol* **14**: 1675–1680.

**Magpie http://www-fp.mcs.anl.gov/~gaasterland/genome.html**

Mannila H, Toivonen H. 1997. Levelwise search and borders of theories in knowledge discovery. *Data Mining Knowledge Discovery* **1**: 241–258.

Marcotte M, Pellegrine M, Thompson MJ, Yeates TO, Eisenberg D. 1999. A combined algorithm for genome-wide prediction of protein function. *Nature* **402**: 83–86.

Mitchell TM. 1997. *Machine Learning*. McGraw-Hill: New York.

Muggleton S. 1991. Inductive logic programming. *New Gen Comput* **8**: 295–318.

Munakata T. 1999. Knowledge discovery. *Comm ACM* **41**: 26–29.

Murzin AG, Brenner SE, Hubbard T, Chothia C. 1995. SCOP: a structural classification of proteins database for the investigation of sequences and structures. *J Mol Biol* **247**: 536–540.

O'Connor CD, Farris M, Hunt LG, Wright JN. 1998. The proteome approach. *Methods Microbiol* **27**: 191–204.

Oliver SG. 1997. Yeast as a navigational aid in genome analysis. *Microbiol UK* **143**: 1483–1487.

Oliver SG, Baganz F. 1998. The yeast genome: systematic analysis of DNA sequence and biological function. In

Copyright © 2000 John Wiley & Sons, Ltd. 

*Genomics: Commercial Opportunities from a Scientific Revolution*, Copping LG, Dixon GK, Livingstone DJ (eds). Bios Scientific Publishing: Oxford; 37–51.

Ontology: The Gene Ontology Consortium. 2000. **http://www.geneontology.org/**

Ouali M, King RD. 2000. Cascaded multiple classifiers for secondary structure prediction. *Protein Sci* **9**: 1162–1176.

Park J, Teichmann SA, Hubbard T, Chothia C. 1997. Intermediate sequences increase the detection of homology between sequences. *J Mol Biol* **273**: 349–354.

Pearson WR, Lipman DJ. 1988. Improved tools for biological sequence comparison. *Proc Natl Acad Sci U S A* **85**: 2444–2448.

Piatetsky-Shapiro G, Frawley W. 1991. *Knowledge Discovery in Databases*. MIT Press: Boston, MA.

**ProtParam_tool**: **http://www.expasy.ch/tools/protparam.html**

Provost F, Fawcett T. 1997. Analysis and visualization of classifier performance: comparison under imprecise class and cost distributions. In *Proceedings of KDD-97*, Heckerman D, Mannila H, Pregibon D (eds). AAAI Press: Menlo Park, CA; 43–48.

Quinlan R. 1993. *C4.5: Programs for Machine Learning*. Morgan Kaufmann: San Mateo.

Rieger KJ, Orlowska G, Kaniak A, Coppee JY, Aljinovic G, Slonimski PP. 1999. Large-scale phenotypic analysis in microtitre plates of mutants with deleted open reading frames from yeast chromosome III: key step between genomic sequencing and protein function. In *Methods in Microbiology 28 (Automation: Genomic and Functional Analysis)*, Crai AG, Joheisel DJ (eds). Academic Press: London; 205–227.

Riley M, Labedan B. 1996. *E. coli* gene products: physiological functions and common ancestries. In *Escherichia coli and Salmonella: Cellular and Molecular Biology*, Neidhardt F *et al.* (eds). American Society for Microbiology: Washington DC; 2118–22002.

**SC_gene_list** **http://www.mips.biochem.mpg.de/proj/yeast/catalogues/index.html**

Tatusov RL, Koonin EV, Lipman DJA. 1997. Genomic perspective on protein families. *Science* **278**: 631–637.

Taylor WR. 1998. Dynamic sequence databank searching with templates and multiple alignments. *J Mol Biol* **280**: 375–406.

**TB_gene_list** **http://www.sanger.ac.uk/Projects/M_tuberculosis/gene_list_full.shtm**

Wilkins MR, Williams KL, Appel RD, Hochstrasser DF. 1997. *Proteome Research: New Frontiers in Functional Genomics*. Springer: Berlin.

*The* **Genomics** *Website at Wiley*

**www.wiley.co.uk/genomics**

The **Genomics** website at Wiley is a new and **DYNAMIC** resource for the genomics community, offering **FREE** special feature articles and new information **EACH MONTH**.

Find out more about *Comparative and Functional Genomics*, and how to view all articles published this year **FREE OF CHARGE**!

Visit the **Library** for hot books in Genomics, Bioinformatics, Molecular Genetics and more.

Click on **Primary Research** for information on all our up-to-the minute journals, including: *Genesis*, *Bioessays, Gene Function and Disease*, and the *Journal of Gene Medicine*.

Let the **Genomics** website at Wiley be your **guide** to genomics-related web sites, manufacturers and suppliers, and a calendar of conferences.

WILEY

1516

Copyright © 2000 John Wiley & Sons, Ltd. 
